# Association of non-high-density lipoprotein cholesterol to high-density lipoprotein cholesterol ratio (NHHR) with cardiovascular mortality in peritoneal dialysis patients: a prospective cohort study

**DOI:** 10.3389/fnut.2026.1827345

**Published:** 2026-07-07

**Authors:** Yuening Zhang, Junjie Liang, Suchun Li, Kexin Ma, Dandan Zhang, Wei Chen, Qinghua Liu, Jing Yu

**Affiliations:** 1 Department of Nephrology, The First Affiliated Hospital, Sun Yat-sen University, Guangzhou, China; 2 Key Laboratory of Nephrology, Ministry of Health and Guangdong Province, Guangzhou, China

**Keywords:** atherosclerosis, cardiovascular mortality, cohort study, non-high-density lipoprotein cholesterol to high-density lipoprotein cholesterol ratio, peritoneal dialysis

## Abstract

**Background:**

The association between non-high-density lipoprotein cholesterol to high-density lipoprotein cholesterol ratio (NHHR) and prognosis in patients with end-stage renal disease (ESRD) remains controversial. The aim of this study is to explore the association of NHHR with cardiovascular disease (CVD) mortality as well as all-cause mortality in peritoneal dialysis (PD) patients.

**Methods:**

In this single-center prospective observational cohort study, 1,616 individuals undergoing PD were recruited with a median follow-up duration of 47.6 months. The association of NHHR with overall CVD mortality, as well as atherosclerotic and non-atherosclerotic subtypes, was assessed using competing risk regression models. Additionally, multivariable Cox proportional hazards regression was employed to evaluate the relationship between NHHR and all-cause mortality. Critical baseline covariates were adjusted in these multivariable models.

**Results:**

In the end, 552 patients (34.2%) died, of whom 264 (47.8%) died from CVD mortality, corresponding to incidence rates of 71.18 and 34.04 per 1,000 person-years, respectively. In multivariable analyses, competing risk regression model showed no significant association between NHHR and overall CVD mortality. However, each 1- standard deviation (SD) increase in NHHR level was associated with a 35% higher risk of atherosclerotic CVD mortality (sub-distribution hazard ratio [SHR] 1.35, 95% Confidence interval [CI] 1.17–1.56), and a 27% lower risk of non-atherosclerotic CVD mortality (SHR 0.73, 95% CI 0.58–0.93), respectively. Cox regression model indicated that each 1-SD increase in NHHR level was independently associated with a 16% increase in all-cause mortality (HR 1.16, 95% CI 1.06–1.26). Quartile-based analysis reinforced these findings.

**Conclusion:**

An elevated NHHR level conferred an increased risk of atherosclerotic CVD and all-cause mortality, yet showed a paradoxical association with reduced non-atherosclerotic CVD mortality in PD patients.

## 1 Introduction

Cardiovascular disease (CVD) is the leading cause of death among patients with end-stage renal disease (ESRD) (1). Approximately 50% of deaths in dialysis-dependent patients are reportedly attributable to CVD (2), with heart failure and sudden cardiac death being the most common manifestations (3). In the general population, higher non-high-density lipoprotein cholesterol (Non-HDL-C) and lower high-density lipoprotein cholesterol (HDL-C) independently raise risks of CVD events and all-cause mortality (4, 5). However, this linear lipid-outcome association complicates with declining renal function (6). Among patients with non-dialysis-dependent chronic kidney disease (NDD-CKD), lower total cholesterol (TC) and low-density lipoprotein cholesterol (LDL-C) correspond to higher risks of CVD events and all-cause mortality (6). This reverse epidemiological phenomenon is mainly mediated by malnutrition-inflammation-cachexia syndrome (MICS) (6). This conflicting pattern is more prominent and contentious in ESRD patients. Uremic toxins inhibit lipoprotein and hepatic lipases, impairing clearance of triglyceride-rich lipoproteins (7). Meanwhile, decreased apolipoprotein A-I synthesis and downregulated lecithin: cholesterol acyltransferase (LCAT) not only lowers the circulating HDL-C levels but also transforms HDL into a dysfunctional, pro-inflammatory particle with compromised antioxidant capacity (7). Consequently, ESRD patients display a distinct atherogenic lipid phenotype marked by hypertriglyceridemia, low HDL-C levels and impaired HDL-C function (7, 8).

One study involving 412 hemodialysis (HD) patients demonstrated that low HDL-C levels were closely associated with CVD incidence (9), while another observational cohort study of 1,764,986 HD patients revealed that high HDL-C levels were significantly linked to poor prognosis (10). Non-HDL-C, which represents the total cholesterol content of all pro-atherogenic lipo-proteins (11), has also yielded conflicting findings. A study of 45,930 HD patients indicated that elevated Non-HDL-C levels were positively correlated with myocardial infarction and cerebral infarction (12). Conversely, another large-scale study paradoxically found that low Non-HDL-C levels were associated with adverse outcomes in HD patients (13). Our previous study identified elevated Non-HDL-C as an independent risk factor for CVD mortality in peritoneal dialysis (PD) patients (14).

NHHR is a readily available composite metric that simultaneously reflects both the burden of pro-atherogenic lipoproteins and the protective capacity of anti-atherogenic lipoproteins, offering a more comprehensive risk assessment by capturing the balance between pathogenic and protective factors. Several studies have established the association between NHHR and mortality in various populations, including patients with sepsis (15), type 2 diabetes (16), and those undergoing percutaneous coronary intervention (17). However, studies on the relationship between NHHR and prognosis in ESRD patients remain limited and inconsistent. A study has shown that NHHR can serve as an effective predictor for stroke risk in HD patients (18). In contrast, a prospective cohort study of 259 HD patients found no significant predictive value of NHHR for CVD mortality (19). Regarding PD patients, only two retrospective cohort studies (20, 21), one of which had a small sample size, have indicated that NHHR predicts all-cause mortality (22). Therefore, this study aims to investigate the association between NHHR and both CVD outcomes and all-cause mortality in a large cohort of PD patients.

## 2 Materials and methods

### 2.1 Study design and population

Between early 2006 and late 2013, we conducted a prospective follow-up of 1,882 individuals diagnosed with ESRD who initiated PD treatment at the First Affiliated Hospital of Sun Yat-sen University. Patients were ineligible if they had a history of hemodialysis exceeding 90 days, previous failed renal transplants, or active malignancies. Additionally, those with missing baseline lipid data were excluded from the final analysis. Exclusion conditions included a history of HD lasting 3 months or more, presence of active malignancy, previous unsuccessful kidney transplantation, or absence of lipid profile data. The final study population consisted of 1,616 individuals, with follow-up continuing through December 31, 2021. The study was conducted in accordance with the Declaration of Helsinki and received ethical clearance from the hospital’s institutional review board. All participants provided written informed consent prior to inclusion.

### 2.2 Data collection and measurements

Upon hospital entry, we collected demographic details and pre-existing medical conditions. The diagnosis of diabetes was established based on standardized criteria or ongoing administration of antidiabetic drugs or insulin. Prior CVD events were identified through documented records of angina, myocardial infarction, heart failure, coronary revascularization procedures, or cerebrovascular accidents (17). Hypertension was characterized as persistent blood pressure readings exceeding 140/90 mmHg or current antihypertensive drug use.

Laboratory tests were conducted within 3 months following the initiation of PD. Lipid parameters, comprising TC, triglycerides (TG), LDL-C, HDL-C, alongside other biochemical indicators, were assayed at the First Affiliated Hospital of Sun Yat-sen University. Laboratory measures employed standardized protocols (23). Additional clinical metrics such as body mass index (BMI), blood pressure, and pharmaceutical regimens were documented concurrently.

Renal reserve was assessed via the estimated glomerular filtration rate (eGFR) derived from the Chronic Kidney Disease Epidemiology Collaboration (CKD-EPI) formula (24). Glucose-based peritoneal dialysate solutions (1.5, 2.5%, or 4.25%) were individualized according to clinical requirements.

### 2.3 Follow-up

The primary exposure was the baseline NHHR level, analyzed both as a continuous variable and a categorical variable divided into quartiles (Q1–Q4). Overall CVD mortality was defined as the primary outcome, and all-cause mortality as the secondary outcome. CVD mortality was further classified into atherosclerotic CVD and non-atherosclerotic CVD mortality based on underlying etiology, in accordance with the standard definition from the American Heart Association/American College of Cardiology (AHA/ACC) (25). Atherosclerotic CVD mortality was defined as death caused by coronary heart disease (including acute myocardial infarction, sudden cardiac death secondary to coronary atherosclerosis, ischemic heart failure, and fatal events during invasive procedures for atherosclerotic disease), ischemic stroke, peripheral arterial disease, or aortic atherosclerotic events. Correspondingly, non-atherosclerotic CVD mortality included deaths from hemorrhagic stroke, non-ischemic heart failure, primary arrhythmia, valvular or pericardial heart disease, unexplained sudden death, pulmonary embolism, or other cardiovascular etiologies unrelated to atherosclerosis.

All death events were jointly adjudicated by board-certified physicians at our center. Adjudication decisions were based on complete original source documents, including official death certificates, full inpatient medical records, imaging reports (coronary angiography, cranial CT/MRI, vascular ultrasound, etc.), and etiological laboratory test results. For out-of-hospital deaths without available clinical examination data, telephone interviews with family members were conducted to ascertain the circumstances of death. The cause of death was subsequently determined by study physicians via comprehensive assessment of perimortem signs and symptoms, past medical history, recent health status, and family member accounts (14, 26).

A dedicated follow-up team consisting of physicians and trained nurses performed monthly follow-ups via telephone or face-to-face visits to track patient status and modify treatment regimens when needed. Patients were also scheduled for quarterly revisits to the center for thorough clinical evaluation (17). Censoring criteria: Patients receiving regular PD until the study end date were censored. Those switching to HD, undergoing kidney transplantation, transferring to other centers, or lost to follow-up were also censored.

### 2.4 Statistical analysis

Participants were stratified into quartiles based on the level of NHHR, quartile 1 (Q1), <2.36; quartile 2, 2.36–3.13; quartile 3 (Q3), 3.13–4.08; quartile 4 (Q4), >4.08, with the first quartile as the reference group. Similar quartile categorization was applied to Non-HDL-C and HDL-C alone. Continuous variables were assessed for normality; those with a normal distribution are summarized as mean ± SD, while skewed data are reported as medians (interquartile ranges). For categorical variables, counts and percentages were utilized. To evaluate differences of variables among groups, analysis of the variance was used for normally distributed continuous variables, Kruskal-Wallis test was chosen for non-normally distributed continuous variables, and weighted chi-square test was applied to categorical variables.

Crude incidence rates for overall CVD and all-cause mortality were calculated as the total number of death events divided by the cumulative follow-up person-years, and expressed as event rates per 1,000 person-years. The cumulative incidence function (CIF) curve was used to assess the risk of overall CVD mortality, as well as atherosclerotic and non-atherosclerotic CVD mortality, with the Fine-Gray test employed as the statistical method (27). We applied Kaplan–Meier survival curve to analyze the association between NHHR and all-cause mortality, using the Log-Rank test for statistical comparison. The relationship between NHHR and the primary and secondary outcomes was visualized using restricted cubic splines with five knots, a widely used statistical method designed to flexibly fit and detect potential non-linear associations between continuous variables and clinical endpoints without imposing a rigid linear assumption.

Due to competing risks between CVD mortality and other causes of death, as well as between atherosclerotic and non-atherosclerotic CVD mortality, we performed multiple competing risk regression analysis to evaluate the association of NHHR with these outcomes. The results are expressed as SHRs with 95% CIs. The SHR estimates adjust for competing events, which could introduce bias in conventional survival analysis. Additionally, Cox proportional hazards regression was used to examine the independent association between NHHR and all-cause mortality, with results reported as hazard ratios (HRs) and 95% CIs. In the multivariable regression analysis, model 1 was adjusted for age and sex, model 2 was adjusted for model 1 plus diabetes, prior CVD events, BMI, and systolic blood pressure (SBP), model 3 was adjusted for model 2 plus hemoglobin, serum albumin (ALB), hypersensitive C-reactive protein (hs-CRP), and eGFR, model 4 was adjusted for model 3 plus statin use.

Subgroup analyses were performed according to relevant clinical parameters, with results visualized via forest plots. A two-sided *p*-value <0.05 was considered statistically significant. All analyses were performed using SPSS version 26.0 and Stata version 14.0.

## 3 Results

### 3.1 Population

**Figure 1:**
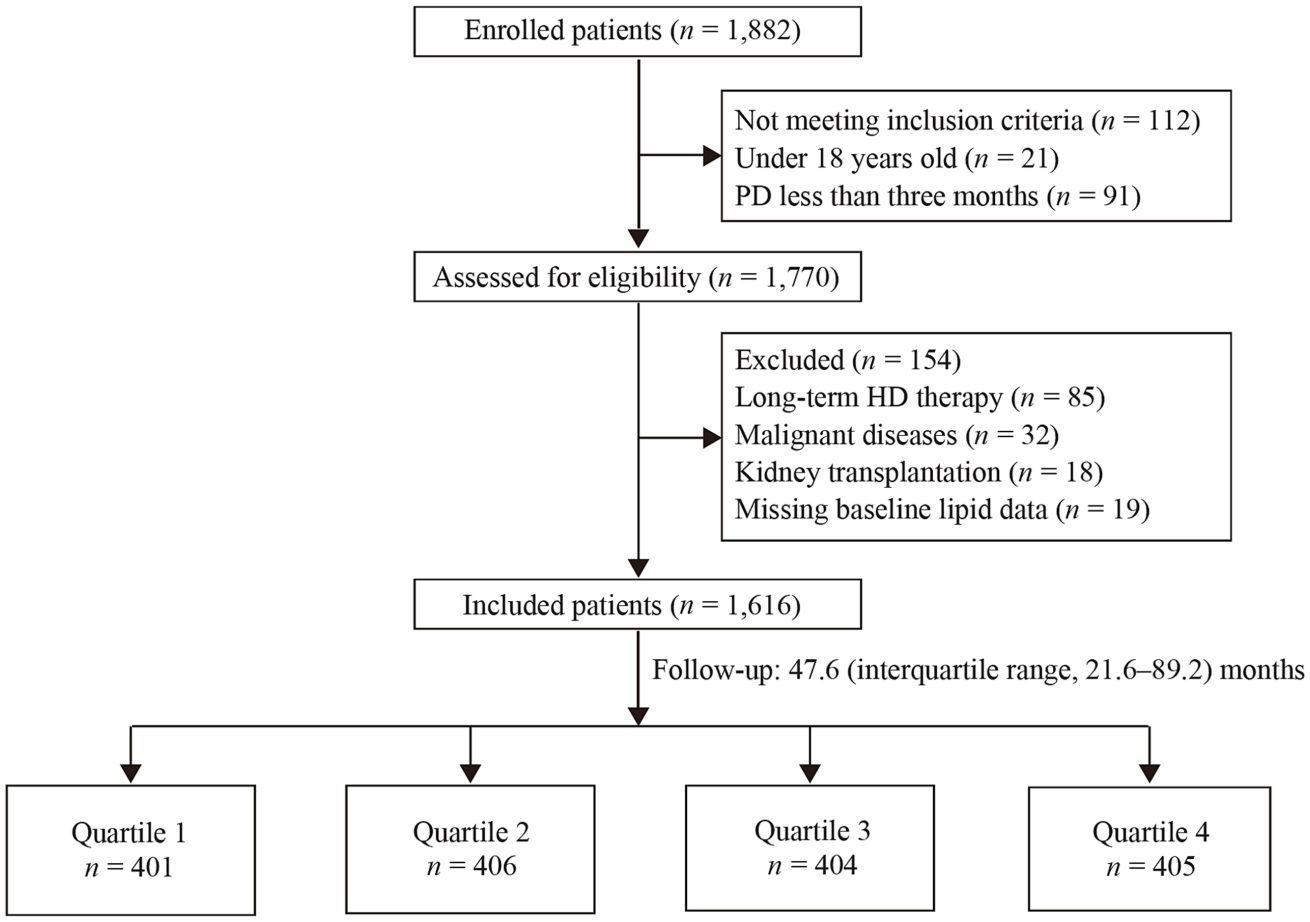
Flow chart for the study. HD, hemodialysis; PD, peritoneal dialysis.

A total of 1,882 patients were screened from January 1st, 2006 to December 31st, 2013. Among them, 266 patients were excluded. Finally, 1,616 patients were enrolled in the final analysis, and were categorized into quartiles based on the NHHR, with 401, 406, 404, and 405 patients in each group, respectively, which was shown in Figure 1. Baseline characteristics of PD patients stratified by quartiles of NHHR were presented in Table 1. The mean age of the enrolled patients was 47.5 ± 15.2 years, and 966 (59.8%) were male. A total of 415 patients (25.7%) had a history of diabetes, and 596 patients (36.9%) had a history of cardiovascular events. The median NHHR was 3.13 (interquartile range: 2.36–4.08). Patients with higher NHHR were generally older and exhibited higher proportions of diabetes, and elevated levels of BMI, hs-CRP, TC, TG, LDL-C, and Non-HDL-C, while showing lower levels of HDL-C.

**Table 1:** Baseline characteristics of the study population.

Covariates	Total (*n* = 1,616)	Quartiles of NHHR				*p*-value
		Q1 (<2.36) (*n* = 401)	Q2 (2.36–3.13) (*n* = 406)	Q3 (3.13–4.08) (*n* = 404)	Q4 (>4.08) (*n* = 405)	
Age (years)	47.5 ± 15.2	45.1 ± 14.9	46.0 ± 15.3	47.8 ± 14.9	51.0 ± 15.1	<0.001
Male *n* (%)	966 (59.8)	220 (54.9)	250 (61.6)	260 (64.4)	236 (58.3)	0.037
Diabetes *n* (%)	415 (25.7)	88 (21.9)	82 (20.2)	87 (21.5)	158 (39.0)	<0.001
Prior CVD events *n* (%)	596 (36.9)	139 (34.7)	147 (36.2)	144 (35.6)	166 (41.0)	0.248
Hypertension *n* (%)	1,439 (89.0)	358 (89.3)	357 (87.9)	353 (87.4)	371 (91.6)	0.220
BMI (kg/m^2^)	21.6 ± 3.1	20.5 ± 2.7	21.2 ± 2.9	22.0 ± 3.1	22.8 ± 3.3	<0.001
SBP (mmHg)	136.5 ± 19.8	136.8 ± 19.5	138.2 ± 20.6	134.2 ± 19.2	136.9 ± 19.6	0.029
DBP (mmHg)	84.7 ± 14.4	85.9 ± 14.8	86.1 ± 14.6	84.2 ± 13.6	82.8 ± 14.4	0.003
Hemoglobin (g/L)	105.0 ± 21.2	102.6 ± 22.1	105.3 ± 21.5	108.2 ± 20.1	103.8 ± 20.8	0.001
Serum albumin (g/L)	37.3 ± 5.2	36.8 ± 5.0	37.3 ± 5.1	38.0 ± 4.9	37.0 ± 5.5	0.005
Hs-CRP (mg/L)	1.75 [0.64–5.74]	1.04 [0.40–3.54]	1.43 [0.66–4.90]	1.78 [0.68–4.92]	3.55 [1.18–9.56]	<0.001
eGFR (mL/min/1.73 m^2^)	6.8 ± 3.1	6.8 ± 2.9	6.9 ± 3.7	6.9 ± 3.0	6.8 ± 2.9	0.863
TC (mg/dL)	196.5 ± 51.4	172.6 ± 41.1	188.6 ± 44.7	198.7 ± 41.7	225.9 ± 60.3	<0.001
TG (mg/dL)	124.8 [89.4–177.0]	84.1 [61.9–108.0]	109.7 [84.1–138.1]	138.1 [108.8–183.0]	200.0 [150.4–283.2]	<0.001
HDL-C (mg/dL)	47.6 ± 14.9	60.8 ± 15.4	50.5 ± 12.0	43.6 ± 9.4	35.6 ± 8.9	<0.001
LDL-C (mg/dL)	113.3 ± 38.7	92.8 ± 26.4	111.9 ± 31.0	119.0 ± 34.0	129.5 ± 49.8	<0.001
Non-HDL-C (mg/dL)	148.9 ± 48.0	111.8 ± 29.0	138.1 ± 33.2	155.1 ± 32.8	190.3 ± 54.4	<0.001
NHHR	3.13 [2.36–4.08]	1.94 [1.65–2.14]	2.72 [2.55–2.95]	3.54 [3.33–3.80]	5.09 [4.49–5.87]	<0.001
Statin use *n* (%)	236 (14.6)	60 (15.0)	54 (13.3)	53 (13.1)	69 (17.0)	0.357

Normally distributed variables are presented as means ± standard deviations (SDs), while non-normally distributed variables are reported as medians and 25th–75th percentiles. Categorical variables are expressed as frequencies and percentages. Unit conversion factors: TC, HDL-C, and LDL-C were converted from mg/dL to mmol/L by multiplying by 0.0259; TG was converted from mg/dL to mmol/L by multiplying by 0.0113. BMI, body mass index; CVD, cardiovascular disease; DBP, diastolic blood pressure; eGFR, estimated glomerular filtration rate; HDL-C, high-density lipoprotein cholesterol; Hs-CRP, hypersensitive C-reactive protein; LDL-C, low-density lipoprotein cholesterol; Non-HDL-C, non-high-density lipoprotein cholesterol; NHHR, non-high-density lipoprotein cholesterol to high-density lipoprotein cholesterol ratio; Q1–Q4, lowest to highest quartile; SBP, systolic blood pressure; TC, total cholesterol; TG, triglycerides.

Over a median follow-up of 47.6 months, 552 patients (34.2%) died, of whom 264 (47.8%) died from CVD mortality, including 158 cases (59.8%) due to atherosclerotic CV mortality, 106 (40.2%) cases due to non-atherosclerotic CVD mortality, respectively. The overall crude incidence rates for all-cause and CVD mortality were 71.18 and 34.04 per 1,000 person-years, respectively. When stratified by NHHR quartiles, the absolute disease burden for both outcomes was highest in Q4 (Supplementary Table S1). The distribution of clinical outcomes and causes of death was shown in Supplementary Figures S1A,B, respectively.

### 3.2 Independent associated factors for the level of NHHR

A multiple linear regression model was used to explore the independent associated factors of NHHR levels (Table 2). First, a simple linear regression model identified that age (*β* = 0.155, *p* < 0.001), presence of diabetes (*β* = 0.153, *p* < 0.001), BMI (*β* = 0.285, *p* < 0.001), diastolic blood pressure (DBP) (*β* = −0.085, *p* = 0.001), and hs-CRP levels (*β* = 0.185, *p* < 0.001) were associated with the level of NHHR. Subsequently, the multiple linear regression model revealed that age (*β* = 0.081, *p* = 0.002), BMI (*β* = 0.270, *p* < 0.001), and hs-CRP levels (*β* = 0.116, *p* < 0.001) were independent associated factors for the level of NHHR in PD patients.

**Table 2:** Independent associated factors for the level of NHHR.

Risk factors	Simple linear regression		Multiple linear regression	
	Beta (95% CI)	*p*-value	Beta (95% CI)	*p*-value
Age (years)	0.155 (0.003, 0.006)	<0.001	0.081 (0.001, 0.004)	0.002
Sex (female/male)	−0.012 (−0.052, 0.032)	0.635		
Diabetes (yes/no)	0.153 (0.101, 0.194)	<0.001	-	-
Prior CVD events (yes/no)	0.048 (0.000, 0.085)	0.052		
Hypertension (yes/no)	0.038 (−0.015, 0.117)	0.129		
BMI (kg/m^2^)	0.285 (0.032, 0.045)	<0.001	0.270 (0.029, 0.042)	<0.001
SBP (mmHg)	−0.011 (−0.001, 0.001)	0.670		
DBP (mmHg)	−0.085 (−0.004, −0.001)	0.001	-	-
Hemoglobin (g/L)	0.016 (−0.001, 0.001)	0.532		
Serum albumin (g/L)	0.003 (−0.004, 0.004)	0.893		
Hs-CRP (mg/L)	0.185 (0.011, 0.019)	<0.001	0.116 (0.005, 0.013)	<0.001
eGFR (mL/min/1.73 m^2^)	0.012 (−0.005, 0.008)	0.634		
Statin use (yes/no)	0.042 (−0.008, 0.108)	0.093		

BMI, body mass index; CI, confidence interval; CVD, cardiovascular disease; DBP, diastolic blood pressure; eGFR, estimated glomerular filtration rate; Hs-CRP, hypersensitive C-reactive protein; NHHR, non-high-density lipoprotein cholesterol to high-density lipoprotein cholesterol ratio; SBP, systolic blood pressure.

### 3.3 Association of NHHR level with CVD and all-cause mortality

**Figure 2:**
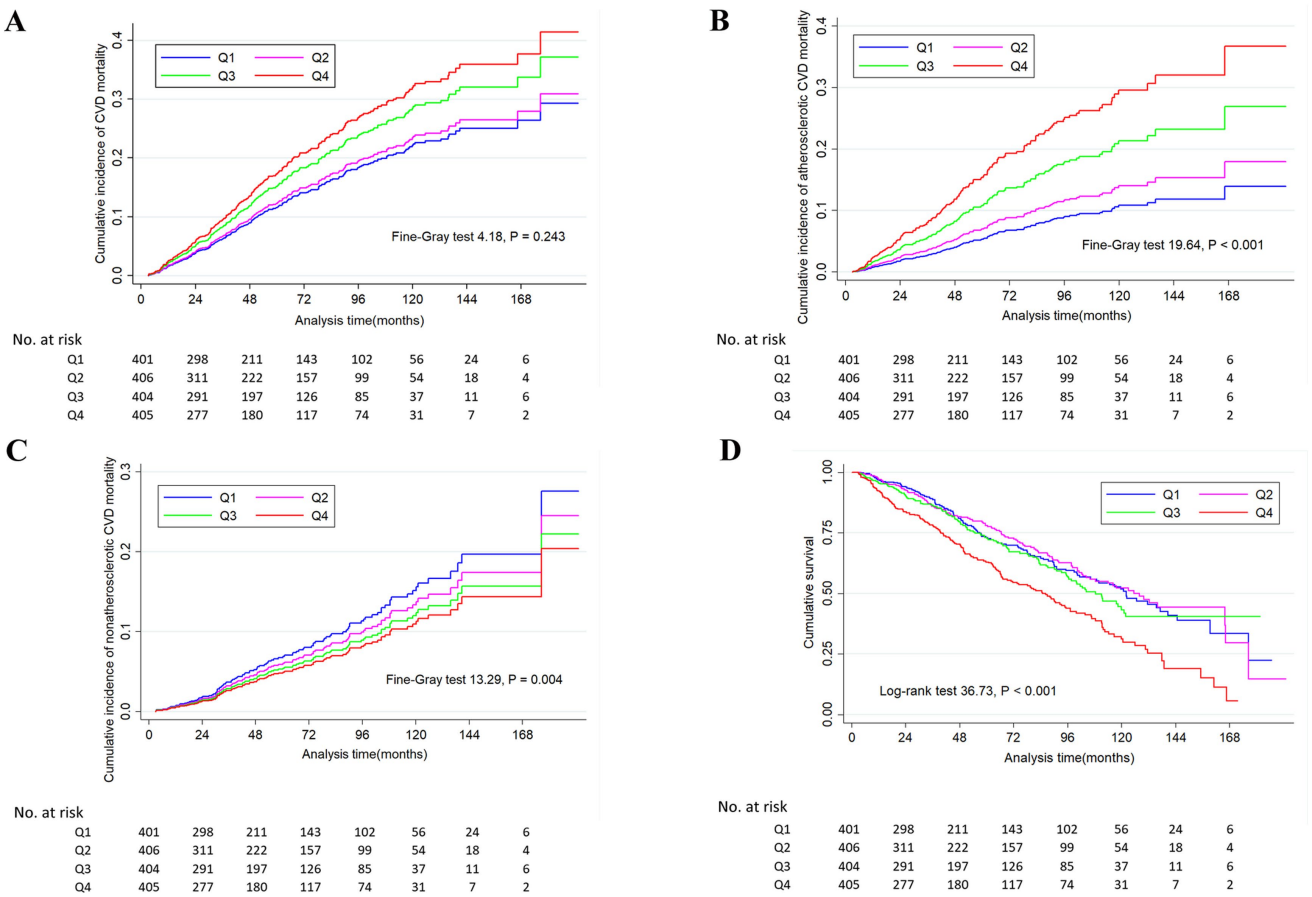
Cumulative incidence function curves and Kaplan–Meier survival curves. Cumulative incidence of overall CVD mortality **(A)**, atherosclerotic CVD mortality **(B)**, and non-atherosclerotic CVD mortality **(C)** in participants categorized by NHHR quartiles. Survival rate of all-cause mortality **(D)** in participants categorized by NHHR quartiles. The number of patients at risk is identical across all survival and competing risk analyses, as all fatal endpoints share the same at-risk population (participants who remained alive and not lost to follow-up at each time point). CVD, cardiovascular disease; Q1–Q4, lowest to highest quartile. NHHR, non-high-density lipoprotein cholesterol to high-density lipoprotein cholesterol ratio.

The results of CIF curves showed that although no significant difference was observed in overall CVD mortality across NHHR quartiles (*p* = 0.243, Figure 2A), stratification by etiology showed that patients in quartile 4 had a significantly higher cumulative incidence of atherosclerotic CVD mortality (*p* < 0.001, Figure 2B), but a lower incidence of non-atherosclerotic CVD mortality (*p* = 0.004, Figure 2C), compared to the lower three quartiles. For the secondary outcome, Kaplan–Meier survival curves indicated higher all-cause mortality in quartile 4 (*p* < 0.001, Figure 2D).

**Figure 3:**
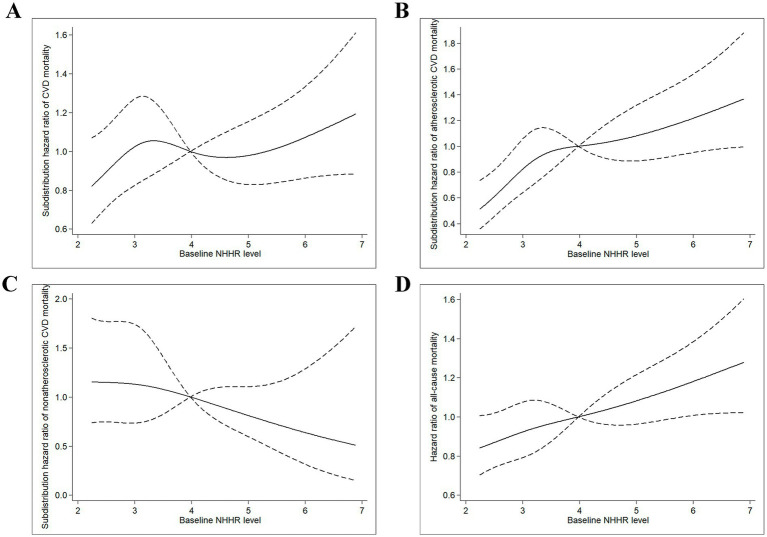
Restricted cubic splines with five knots. Association of NHHR with multivariable-adjusted overall CVD mortality **(A)**, atherosclerotic CVD mortality **(B)**, non-atherosclerotic CVD mortality **(C)**, and all-cause mortality **(D)**. CVD, cardiovascular disease; NHHR, non-high-density lipoprotein cholesterol to high-density lipoprotein cholesterol ratio.

Restricted cubic spline models with five knots further illustrated these trends. NHHR exhibited a nonlinear association with overall CVD mortality (Figure 3A), but displayed an approximately positive linear association with atherosclerotic CVD mortality (Figure 3B), and a negative linear association with non-atherosclerotic CVD mortality (Figure 3C). A positive linear association was also observed between NHHR and all-cause mortality (Figure 3D).

The association between NHHR and the prognosis of PD patients was shown in Table 3. In multivariable analyses, competing risk regression model showed no significant association between NHHR and overall CVD mortality (per 1-SD increase: SHR 1.05, 95% CI 0.92–1.20, *p* = 0.464). However, after etiologic stratification, each 1-SD increase in NHHR level was associated with a 35% higher risk of atherosclerotic CVD mortality (SHR 1.35, 95% CI 1.17–1.56, *p* < 0.001), and a 27% lower risk of non-atherosclerotic CVD mortality (SHR 0.73, 95% CI 0.58–0.93, *p* = 0.012), respectively. Quartile-based analysis reinforced these findings, with the highest quartile showing significantly elevated risk for atherosclerotic CVD mortality (SHR 2.13, 95% CI, 1.22–3.70, *p* = 0.008) and a trend toward reduced risk for non-atherosclerotic CVD mortality (SHR 0.59, 95% CI, 0.33–1.08, *p* = 0.087), compared to the lowest quartile. Similarly, Cox regression model indicated that each 1-SD increase in NHHR was independently associated with a 16% increase in all-cause mortality (HR 1.16, 95% CI 1.06–1.26, *p* = 0.001), with quartile 4 showing significantly elevated risk compared to quartile 1 (HR 1.33, 95% CI, 1.02–1.72, *p* = 0.033).

**Table 3:** Association of NHHR with CVD, atherosclerotic, non-atherosclerotic CVD and all-cause mortality.

	Per 1-SD increase		Q2 (*n* = 406)		Q3 (*n* = 404)		Q4 (*n* = 405)	
	SHR (95% CI)	*p*-value	SHR (95% CI)	*p*-value	SHR (95% CI)	*p-*value	SHR (95% CI)	*p*-value
CVD mortality								
Unadjusted	1.15 (1.04–1.26)	0.005	1.07 (0.74–1.53)	0.730	1.34 (0.94–1.91)	0.107	1.54 (1.09–2.17)	0.013
Model 1	1.07 (0.97–1.19)	0.176	0.98 (0.68–1.42)	0.917	1.23 (0.86–1.75)	0.261	1.21 (0.85–1.72)	0.282
Model 2	1.03 (0.91–1.15)	0.671	0.95 (0.65–1.39)	0.802	1.20 (0.83–1.73)	0.336	1.07 (0.74–1.54)	0.719
Model 3	1.04 (0.91–1.19)	0.584	0.99 (0.66–1.47)	0.948	1.16 (0.78–1.72)	0.455	1.04 (0.70–1.54)	0.843
Model 4	1.05 (0.92–1.20)	0.464	1.02 (0.68–1.53)	0.923	1.20 (0.81–1.79)	0.358	1.09 (0.73–1.61)	0.676
Atherosclerotic CVD mortality								
Unadjusted	1.38 (1.21–1.59)	<0.001	1.32 (0.77–2.25)	0.312	2.09 (1.27–3.45)	0.004	3.06 (1.89–4.93)	<0.001
Model 1	1.43 (1.28–1.61)	<0.001	1.18 (0.68–2.06)	0.550	1.89 (1.13–3.14)	0.014	2.45 (1.51–3.96)	<0.001
Model 2	1.36 (1.19–1.54)	<0.001	1.12 (0.64–1.97)	0.689	1.93 (1.15–3.25)	0.013	2.18 (1.32–3.60)	0.002
Model 3	1.33 (1.16–1.53)	<0.001	1.15 (0.64–2.08)	0.634	1.83 (1.06–3.16)	0.031	1.93 (1.14–3.29)	0.015
Model 4	1.35 (1.17–1.56)	<0.001	1.22 (0.67–2.22)	0.515	1.96 (1.11–3.45)	0.020	2.13 (1.22–3.70)	0.008
Non-atherosclerotic CVD mortality								
Unadjusted	0.80 (0.64–1.00)	0.048	0.87 (0.53–1.44)	0.592	0.78 (0.46–1.32)	0.355	0.71 (0.41–1.23)	0.216
Model 1	0.71 (0.56–0.90)	0.005	0.81 (0.49–1.34)	0.416	0.68 (0.40–1.15)	0.149	0.54 (0.31–0.96)	0.035
Model 2	0.69 (0.54–0.88)	0.002	0.80 (0.47–1.34)	0.390	0.68 (0.39–1.19)	0.181	0.51 (0.29–0.90)	0.021
Model 3	0.74 (0.58–0.94)	0.013	0.90 (0.51–1.58)	0.707	0.74 (0.41–1.35)	0.330	0.60 (0.33–1.09)	0.094
Model 4	0.73 (0.58–0.93)	0.012	0.89 (0.51–1.57)	0.699	0.74 (0.41–1.35)	0.322	0.59 (0.33–1.08)	0.087

	Per 1-SD increase		Q2 (*n* = 406)		Q3 (*n* = 404)		Q4 (*n* = 405)	
	HR (95% CI)	*p*-value	HR (95% CI)	*p*-value	HR (95% CI)	*p*-value	HR (95% CI)	*p*-value
All-cause mortality								
Unadjusted	1.24 (1.16–1.33)	<0.001	0.95 (0.74–1.22)	0.700	1.10 (0.86–1.42)	0.436	1.74 (1.38–2.18)	< 0.001
Model 1	1.20 (1.11–1.30)	<0.001	0.86 (0.67–1.10)	0.231	0.99 (0.77–1.27)	0.948	1.31 (1.04–1.65)	0.020
Model 2	1.17 (1.08–1.27)	<0.001	0.88 (0.68–1.13)	0.306	1.08 (0.83–1.39)	0.579	1.26 (1.00–1.61)	0.055
Model 3	1.15 (1.05–1.26)	0.002	0.96 (0.73–1.25)	0.740	1.15 (0.88–1.51)	0.312	1.29 (1.00–1.67)	0.050
Model 4	1.16 (1.06–1.26)	0.001	0.97 (0.74–1.27)	0.834	1.18 (0.89–1.55)	0.245	1.33 (1.02–1.72)	0.033

We indicated the lowest quartile (Q1) as the reference group (*n* = 401). CI, confidence interval; CVD, cardiovascular disease; HR, hazard ratio; NHHR, non-high-density lipoprotein cholesterol to high-density lipoprotein cholesterol ratio; Q1 to Q4, lowest to highest quartile; SD, standard deviation; SHR, subdistribution hazard ratio. Model 1: Adjusted for age and sex. Model 2: Adjusted for model 1 plus diabetes, prior CVD events, body mass index, and systolic blood pressure. Model 3: Adjusted for model 2 plus hemoglobin, serum albumin, hypersensitive C-reactive protein, and estimated glomerular filtration rate. Model 4: Adjusted for model 3 plus statin use.

Restricted cubic spline models with five knots revealed distinct associations for Non-HDL-C and HDL-C with mortality outcomes. Non-HDL-C showed a positive linear association with overall CVD mortality (Supplementary Figure S2A) and atherosclerotic CVD mortality (Supplementary Figure S2B), but a nonlinear association with non-atherosclerotic CVD mortality (Supplementary Figure S2C). A positive linear association was also observed between Non-HDL-C and all-cause mortality (Supplementary Figure S2D). Multivariate analysis confirmed that elevated Non-HDL-C levels independently increased the risk of overall CVD, atherosclerotic CVD, and all-cause mortality, but not non-atherosclerotic CVD mortality (Supplementary Table S2). In contrast, HDL-C exhibited nonlinear associations across all mortality endpoints, including overall CVD (Supplementary Figure S3A), atherosclerotic CVD (Supplementary Figure S3B), non-atherosclerotic CVD (Supplementary Figure S3C), and all-cause mortality (Supplementary Figure S3D), yet multivariable analyses showed no significant association with any of these outcomes (Supplementary Table S3).

### 3.4 Subgroup analyses

**Figure 4:**
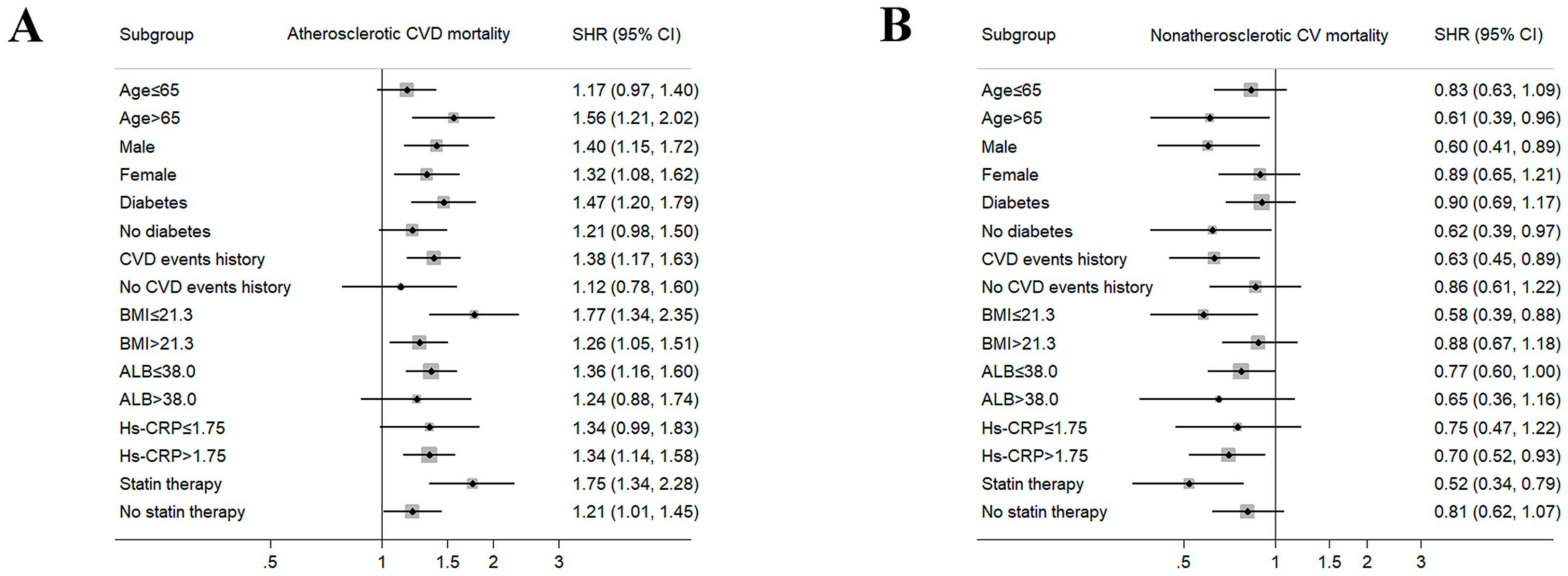
Subgroup analyses. Association between NHHR and atherosclerotic CVD mortality **(A)** and non-atherosclerotic CVD mortality **(B)** using forest plots. ALB, serum albumin; BMI, body mass index; CI, confidence interval; CVD, cardiovascular disease; Hs-CRP, hypersensitive C-reactive protein; SHR, sub-distribution hazard ratio.

Subgroup analyses were performed to assess the robustness of the above associations. As shown in Figure 4A, in subgroups of patients aged >65 years, those with diabetes, prior CVD, albumin ≤38.0 g/L, and hs-CRP >1.75 mg/L, NHHR elevation was significantly associated with higher atherosclerotic CVD mortality. Conversely, as presented in Figure 4B, NHHR elevation was significantly associated with lower risk of non-atherosclerotic CVD mortality in subgroups including those aged >65 years, males, non-diabetics, those with prior CVD, lower BMI, lower albumin, higher hs-CRP, and statin users.

The risk of atherosclerotic CVD mortality significantly increased with elevated Non-HDL-C levels across all subgroups (Supplementary Figure S4A), while no association between Non-HDL-C levels and non-atherosclerotic CVD mortality was observed (Supplementary Figure S4B).

## 4 Discussion

To the best of our knowledge, this is the first large cohort study examining the association between NHHR and both CVD and all-cause mortality in PD patients. Overall, NHHR showed no significant association with total CVD mortality. However, after stratifying CVD mortality into atherosclerotic and non-atherosclerotic subtypes, higher NHHR was linked to increased atherosclerotic CVD mortality but decreased non-atherosclerotic CVD mortality. Elevated NHHR was also independently associated with higher all-cause mortality.

Previous studies on NHHR and CVD mortality in dialysis populations have yielded inconsistent results. In a cohort of 50,118 HD patients, Chang et al. paradoxically found that lower NHHR was significantly associated with elevated CVD mortality (13). This discrepancy is primarily explained by distinct spectra of CVD etiologies between HD and PD populations (23, 28). HD patients predominantly develop non-atherosclerotic CVD events (congestive heart failure, arrhythmia, sudden cardiac death) (23, 28), driven by anemia, calcium-phosphate dysregulation, volume overload, uremic toxins, and electrolyte imbalances (29). These factors induce myocardial interstitial fibrosis and arterial stiffening, ultimately impairing cardiac function (29, 30). In contrast, PD patients have a higher proportion of atherosclerotic CVD events: long-term glucose absorption from dialysate and compensatory hepatic lipoprotein synthesis secondary to peritoneal protein loss raise circulating lipoprotein levels, which infiltrate the vascular intima, promote cholesterol deposition and fibrous plaque formation, and accelerate atherosclerosis (31–33). Concurrent metabolic abnormalities such as hyperinsulinemia further exacerbate endothelial dysfunction and inflammatory activation (31, 32). Consistent with two stratified analyses from the Study of Heart and Renal Protection (SHARP) (34, 35), our study confirmed atherosclerotic CVD as the leading cause of CVD death in PD patients, supporting the biological plausibility of the positive association between NHHR and atherosclerotic CVD mortality. These findings underscore the importance of distinguishing CVD etiologies when evaluating lipid markers and CVD outcomes in ESRD.

The inverse association between NHHR and non-atherosclerotic CVD mortality aligns with prior evidence. Lamprea-Montealegre et al. reported that a higher TG/HDL-C ratio correlated with reduced non-atherosclerotic CVD mortality in 9,270 CKD patients from the SHARP trial, with a more pronounced effect in patients with elevated C-reactive protein (35). We postulate this inverse relationship does not reflect a protective effect of high NHHR, but rather serves as a surrogate for severe malnutrition-inflammation complex syndrome in advanced PD, a well-established driver of non-atherosclerotic CVD mortality.

The independent link between elevated NHHR and all-cause mortality is supported by underlying pathophysiological mechanisms, particularly as infection ranks as the second leading cause of death after CVD in our cohort. As a composite marker, NHHR integrates the pro-inflammatory effects of Non-HDL-C and the immuno-protective functions of HDL-C. Excess Non-HDL-C drives immune dysfunction by inducing T-cell metabolic impairment with reduced mitochondrial respiration (36–39), shifting macrophage polarization toward pro-inflammatory M1 phenotypes (40), and activating the NOD-like receptor family pyrin domain containing 3 (NLRP3) inflammasome via lectin-like oxidized low-density lipoprotein receptor-1 (LOX-1) signaling to promote interleukin-1β (IL-1β) maturation, collectively amplifying oxidative stress and inflammatory cascades (39, 41, 42). Conversely, HDL-C exerts multifaceted immunoprotection: it neutralizes circulating endotoxins via apolipoprotein M (43–45), modulates T-cell receptor activity by maintaining membrane cholesterol homeostasis (46, 47), and exerts anti-inflammatory and antioxidant effects through bioactive components such as sphingosine-1-phosphate (48–52). Taken together, the pro-inflammatory state from elevated Non-HDL-C and impaired immune protection from reduced HDL-C provide a mechanistic basis for the association between higher NHHR and adverse outcomes in PD patients.

Several limitations of this study need to be addressed. First, this single-center cohort only enrolled Chinese patients, restricting the extrapolation of results to other ethnic and geographical populations. Multi-center prospective studies with multi-ethnic cohorts are needed to verify our findings. Second, we solely adopted baseline NHHR values and lacked longitudinal dynamic measurements; fluctuating NHHR during PD may modify prognosis, so serial NHHR testing in prospective trials is required to clarify the prognostic impact of temporal changes. Third, despite extensive covariate adjustment, dynamic confounders such as dialysis regimen modifications, fluctuating inflammatory biomarkers and infection history were not incorporated, potentially causing residual confounding. Future risk models should integrate these key time-varying indicators for more comprehensive risk stratification.

## 5 Conclusion

In summary, we found that elevated NHHR is an independent risk factor for both atherosclerotic CVD mortality and all-cause mortality in PD patients, yet paradoxically associated with reduced non-atherosclerotic CVD mortality. These relationships remained statistically significant after comprehensive covariate adjustment and subgroup analyses. We propose that NHHR may serve as a convenient and practical risk-stratification indicator to guide individualized intensive lipid management and anti-inflammatory interventions. Nevertheless, its actual clinical value remains to be verified by further large-sample, multicenter, interventional randomized controlled trials in the future.

## Data Availability

The datasets presented in this article are not readily available because this data pertains to the privacy of the patients and is not suitable for public disclosure or for being provided without reservation to others. Requests to access the datasets should be directed to Jing Yu, yujing58@mail.sysu.edu.cn.

## Glossary

ALB: serum albumin
ACC: American College of Cardiology
AHA: American Heart Association
BMI: body mass index
CI: confidence interval
CIF: cumulative incidence function
CKD-EPI: chronic kidney disease epidemiology collaboration
CVD: cardiovascular disease
DBP: diastolic blood pressure
eGFR: estimated glomerular filtration rate
ESRD: end-stage renal disease
HD: hemodialysis
HDL-C: high-density lipoprotein cholesterol
HR: hazard ratio
Hs-CRP: hypersensitive C-reactive protein
IL-1β: interleukin-1β
LCAT: cholesterol acyltransferase
LDL-C: low-density lipoprotein cholesterol
LOX-1: lectin-like oxidized low-density lipoprotein receptor-1
MICS: malnutrition-inflammation-cachexia syndrome
NDD-CKD: non-dialysis-dependent chronic kidney disease
NLRP3: NOD-like receptor family pyrin domain containing 3
Non-HDL-C: non-high-density lipoprotein cholesterol
NHHR: non-high-density lipoprotein cholesterol to high-density lipoprotein cholesterol ratio
PD: peritoneal dialysis
Q1–Q4: lowest to highest quartile
SBP: systolic blood pressure
SD: standard deviation
SHARP: study of heart and renal protection
SHR: sub-distribution hazard ratio
TC: total cholesterol
TG: triglycerides
