# Supplementary figures and images for: Association of non-high-density lipoprotein cholesterol to high-density lipoprotein cholesterol ratio (NHHR) with cardiovascular mortality in peritoneal dialysis patients: a prospective cohort study
Source: Front Nutr. 2026 Jul 7;13:1827345. doi: 10.3389/fnut.2026.1827345 (PMC13385109; doi:10.3389/fnut.2026.1827345)

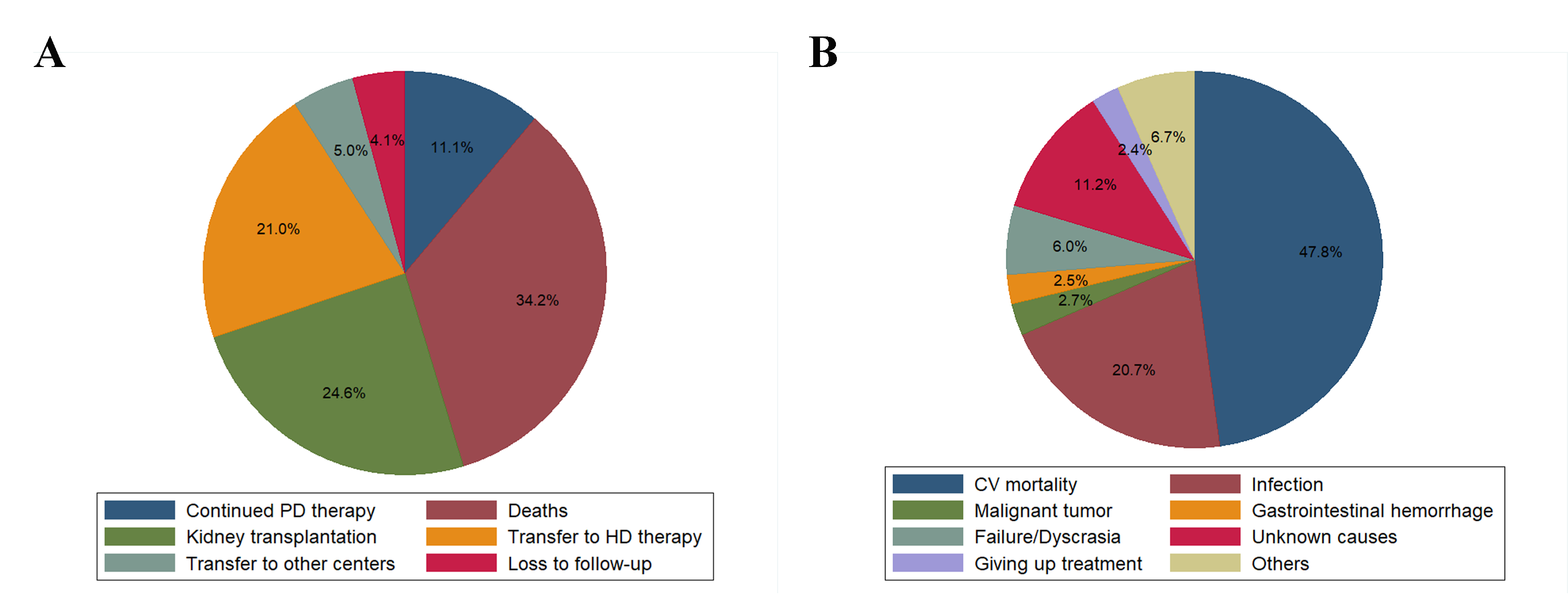

Supplement: Supplementary file 1 [file Image_1.TIF]

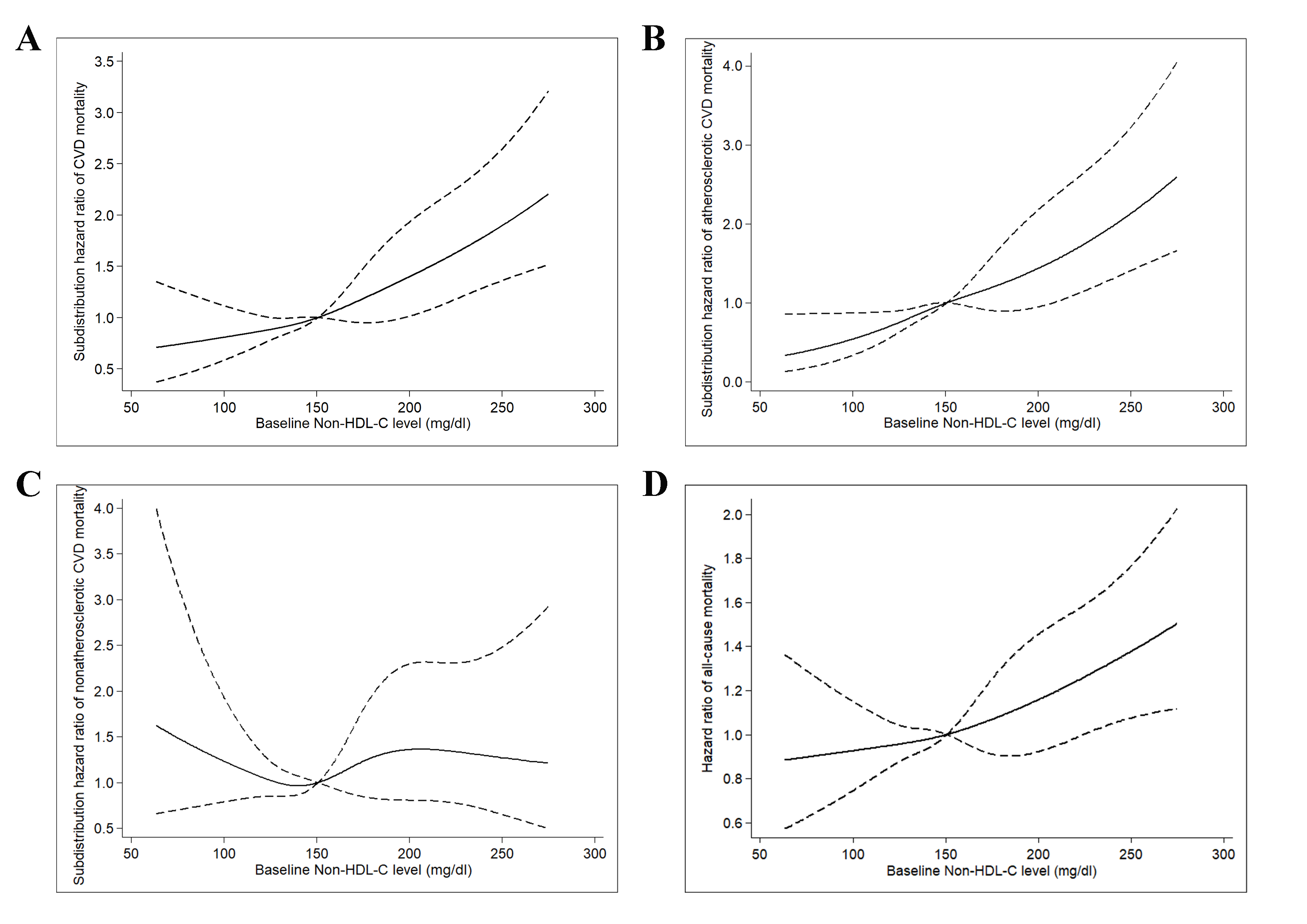

Supplement: Supplementary file 2 [file Image_2.TIF]

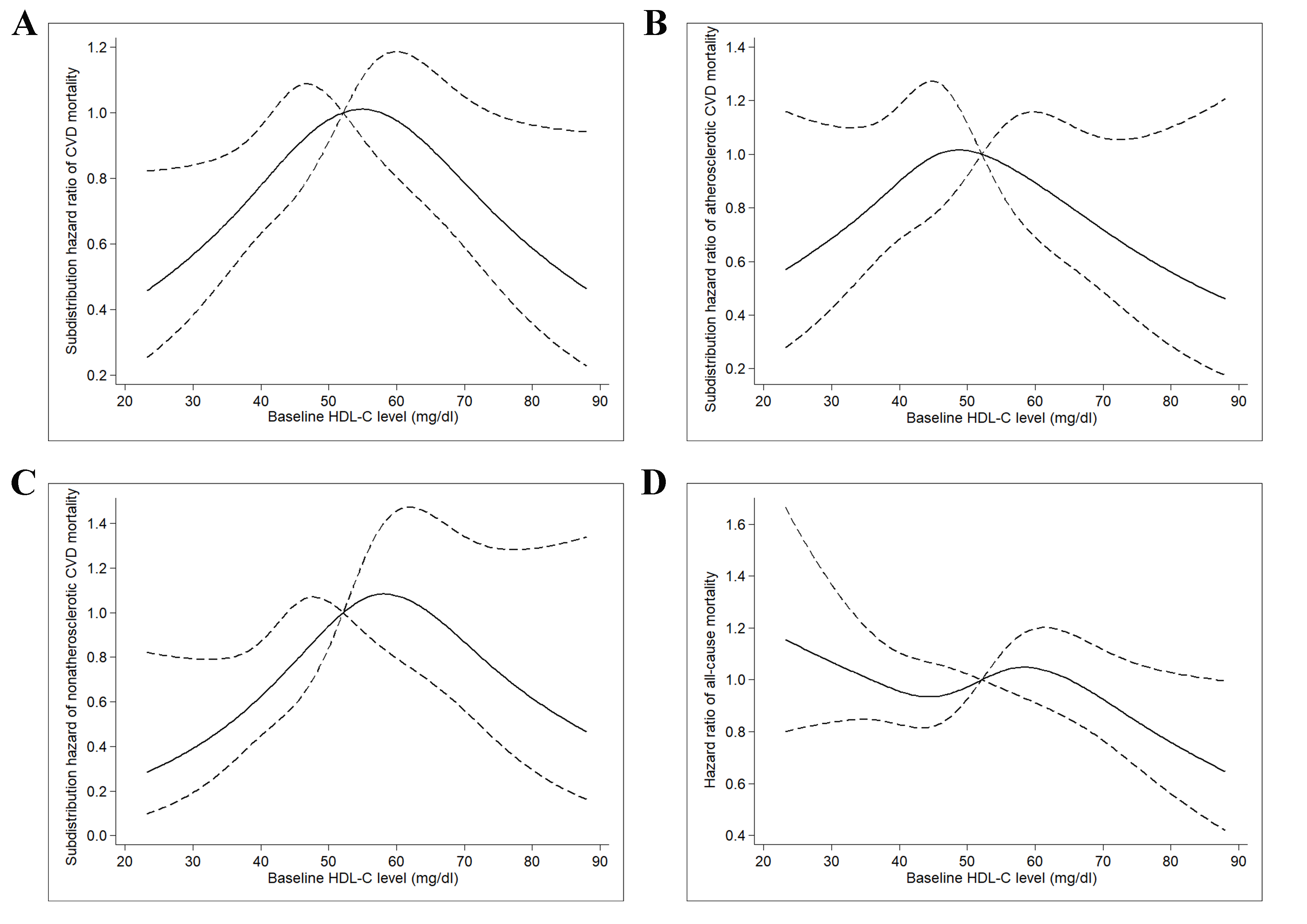

Supplement: Supplementary file 3 [file Image_3.TIF]

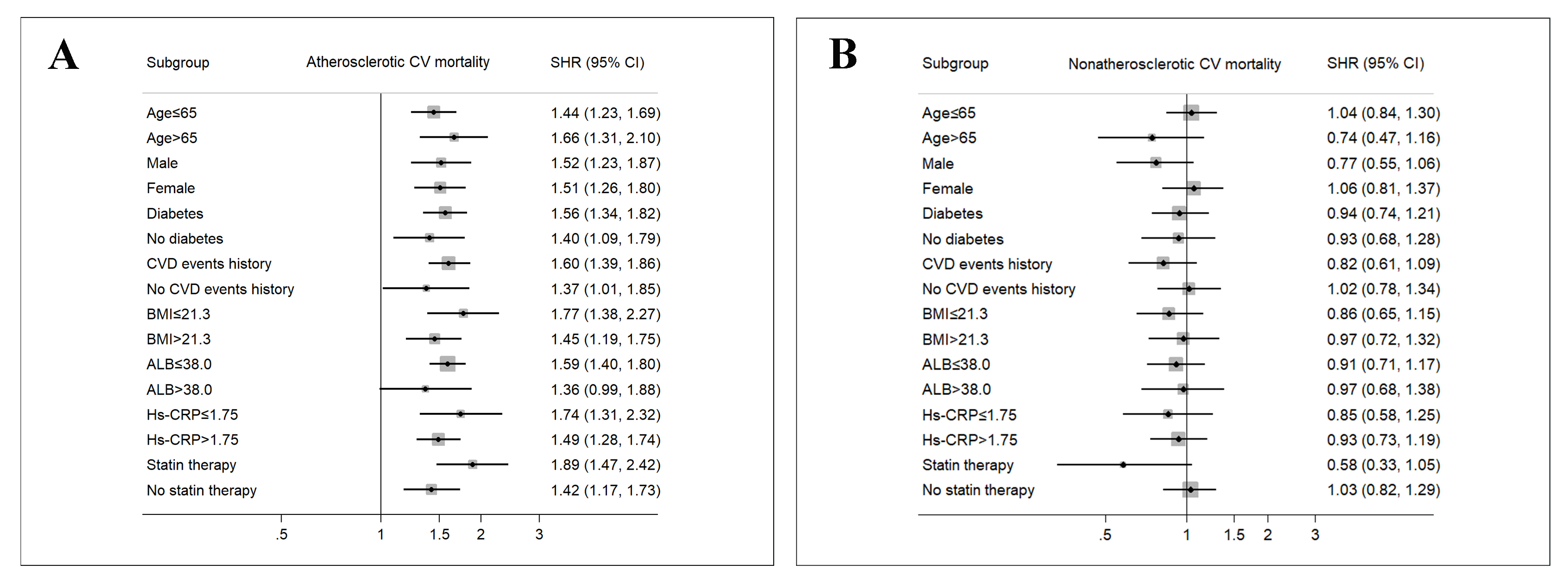

Supplement: Supplementary file 4 [file Image_4.TIF]
