# Supplementary material for: Association of non-high-density lipoprotein cholesterol to high-density lipoprotein cholesterol ratio (NHHR) with cardiovascular mortality in peritoneal dialysis patients: a prospective cohort study
Source: Front Nutr. 2026 Jul 7;13:1827345. doi: 10.3389/fnut.2026.1827345 (PMC13385109; doi:10.3389/fnut.2026.1827345)
Supplement: Supplementary file 5 [file Table_1.PDF]

Table S1 All-cause and CVD Mortality Event Rate

NHHR Quartiles	Patients (n)	Total Follow-up (person-years)	All-cause Mortality Events (n)	All-cause Mortality Rate (per 1,000 person-years)	CVD Mortality Events (n)	CVD Mortality Rate (per 1,000 person-years)
**Q1**	401	2029.48	125	61.59	55	27.10
**Q2**	406	2088.80	122	58.41	60	28.72
**Q3**	404	1876.39	125	66.62	68	36.24
**Q4**	405	1760.78	180	102.23	81	46.00
**Total**	1,616	7755.45	552	71.18	264	34.04
