# Supplementary material for: Association of non-high-density lipoprotein cholesterol to high-density lipoprotein cholesterol ratio (NHHR) with cardiovascular mortality in peritoneal dialysis patients: a prospective cohort study
Source: Front Nutr. 2026 Jul 7;13:1827345. doi: 10.3389/fnut.2026.1827345 (PMC13385109; doi:10.3389/fnut.2026.1827345)
Supplement: Supplementary file 6 [file Table_2.PDF]

Table S2. Association of Non-HDL-C With CVD, Atherosclerotic, Nonatherosclerotic CVD And All-cause Mortality

	Per 1-SD increase		Q2 (n = 396)		Q3 (n = 409)		Q4 (n = 402)	
	**SHR (95% CI)**	***P* value**	**SHR (95% CI)**	***P* value**	**SHR (95% CI)**	***P* value**	**SHR (95% CI)**	***P* value**
**CVD mortality**								
Unadjusted	1.31 (1.16 – 1.47)	< 0.001	1.14 (0.79 – 1.66)	0.482	1.11 (0.77 – 1.59)	0.585	1.81 (1.29 – 2.54)	0.001
Model 1	1.30 (1.15 – 1.46)	< 0.001	1.24 (0.85 – 1.80)	0.270	1.02 (0.70 – 1.50)	0.906	1.85 (1.30 – 2.63)	0.001
Model 2	1.27 (1.13 – 1.43)	< 0.001	1.21 (0.82 – 1.78)	0.337	1.11 (0.76 – 1.63)	0.591	1.75 (1.22 – 2.53)	0.003
Model 3	1.34 (1.19 – 1.51)	< 0.001	1.31 (0.86 – 2.01)	0.212	1.20 (0.78 – 1.82)	0.406	2.05 (1.38 – 3.05)	< 0.001
Model 4	1.36 (1.21 – 1.52)	< 0.001	1.42 (0.92 – 2.19)	0.116	1.25 (0.82 – 1.92)	0.298	2.21 (1.48 – 3.31)	< 0.001
**Atherosclerotic CVD mortality**								
Unadjusted	1.55 (1.37 – 1.75)	< 0.001	1.21 (0.71 – 2.07)	0.480	1.58 (0.97 – 2.59)	0.068	2.68 (1.68 – 4.27)	< 0.001

Model 1	1.51 (1.32 – 1.72)	< 0.001	1.27 (0.74 – 2.18)	0.392	1.40 (0.84 – 2.34)	0.197	2.57 (1.57 – 4.18)	< 0.001
Model 2	1.51 (1.34 – 1.71)	< 0.001	1.38 (0.79 – 2.44)	0.260	1.68 (0.99 – 2.86)	0.056	2.64 (1.57 – 4.44)	< 0.001
Model 3	1.53 (1.34 – 1.73)	< 0.001	1.57 (0.86 – 2.89)	0.144	1.67 (0.93 – 2.97)	0.083	2.90 (1.65 – 5.09)	< 0.001
Model 4	1.54 (1.36 – 1.75)	< 0.001	1.82 (0.94 – 3.53)	0.077	1.86 (1.01 – 3.42)	0.046	3.36 (1.81 – 6.21)	< 0.001
**Nonatherosclerotic CVD mortality**								
Unadjusted	0.93 (0.75 – 1.14)	0.487	1.04 (0.61 – 1.76)	0.885	0.71 (0.40 – 1.25)	0.236	1.01 (0.61 – 1.70)	0.959
Model 1	0.87 (0.70 – 1.08)	0.211	1.05 (0.62 – 1.77)	0.863	0.62 (0.34 – 1.11)	0.109	0.88 (0.52 – 1.49)	0.630
Model 2	0.83 (0.67 – 1.04)	0.102	1.03 (0.60 – 1.75)	0.913	0.59 (0.32 – 1.09)	0.091	0.79 (0.47 – 1.34)	0.382
Model 3	0.92 (0.75 – 1.13)	0.423	1.12 (0.61 – 2.03)	0.720	0.68 (0.35 – 1.33)	0.261	1.03 (0.58 – 1.82)	0.930

Model 4	0.92 (0.75 – 1.13)	0.419	1.12 (0.62 – 2.01)	0.711	0.68 (0.35 – 1.33)	0.262	1.03 (0.59 – 1.80)	0.924
	**Per 1-SD increase HR (95% CI)**	***P* value**	**HR (95% CI)**	***P* value**	**HR (95% CI)**	***P* value**	**HR (95% CI)**	***P* value**
**All-cause mortality**								
Unadjusted	1.20 (1.10 – 1.30)	< 0.001	0.99 (0.77 – 1.27)	0.924	1.12 (0.88 – 1.43)	0.353	1.46 (1.16 – 1.84)	0.001
Model 1	1.09 (1.00 – 1.19)	0.042	1.03 (0.80 – 1.33)	0.800	0.87 (0.68 – 1.11)	0.268	1.18 (0.93 – 1.50)	0.172
Model 2	1.08 (1.00 – 1.18)	0.066	1.06 (0.82 – 1.36)	0.673	0.94 (0.74 – 1.21)	0.644	1.15 (0.90 – 1.47)	0.255
Model 3	1.14 (1.04 – 1.24)	0.005	1.23 (0.93 – 1.63)	0.138	0.95 (0.73 – 1.25)	0.727	1.34 (1.03 – 1.75)	0.030
Model 4	1.14 (1.04 – 1.25)	0.004	1.28 (0.97 – 1.70)	0.086	0.98 (0.74 – 1.28)	0.863	1.39 (1.06 – 1.82)	0.018

Note: We indicated the lowest quartile (Q1) as the reference group (n = 409).

Abbreviations: CI, confidence interval; CVD, cardiovascular disease; HR, hazard ratio; Non-HDL-C, non-high-density lipoprotein cholesterol; Q1 to Q4, lowest to highest quartile; SD, standard deviation; SHR, subdistribution hazard ratio.

Model 1: Adjusted for age and sex.

Model 2: Adjusted for model 1 plus diabetes, prior CVD events, body mass index, and systolic blood pressure.

Model 3: Adjusted for model 2 plus hemoglobin, serum albumin, hypersensitive C-reactive protein, and estimated glomerular filtration rate.

Model 4: Adjusted for model 3 plus statin use.
