# Supplementary material for: Association of non-high-density lipoprotein cholesterol to high-density lipoprotein cholesterol ratio (NHHR) with cardiovascular mortality in peritoneal dialysis patients: a prospective cohort study
Source: Front Nutr. 2026 Jul 7;13:1827345. doi: 10.3389/fnut.2026.1827345 (PMC13385109; doi:10.3389/fnut.2026.1827345)
Supplement: Supplementary file 7 [file Table_3.PDF]

Table S3. Association of HDL-C with CVD, Atherosclerotic, Nonatherosclerotic CVD And All-cause Mortality

	Q1 (n = 406)		Q2 (n = 411)		Q4 (n = 417)	
	SHR (95% CI)	*P* value	SHR (95% CI)	*P* value	SHR (95% CI)	*P* value
**CVD mortality**						
Unadjusted	0.86 (0.60 – 1.24)	0.427	1.49 (1.08 – 2.06)	0.015	0.90 (0.64 – 1.28)	0.571
Model 1	0.68 (0.47 – 0.98)	0.040	1.33 (0.96 – 1.84)	0.089	0.87 (0.61 – 1.25)	0.447
Model 2	0.62 (0.43 – 0.90)	0.013	1.34 (0.97 – 1.86)	0.077	0.98 (0.68 – 1.42)	0.914
Model 3	0.50 (0.34 – 0.76)	0.001	1.26 (0.90 – 1.77)	0.183	0.98 (0.66 – 1.44)	0.905
Model 4	0.50 (0.33 – 0.76)	0.001	1.27 (0.90 – 1.80)	0.170	0.97 (0.66 – 1.44)	0.889
**Atherosclerotic CVD mortality**						
Unadjusted	1.27 (0.80 – 2.03)	0.316	1.91 (1.25 – 2.93)	0.003	0.83 (0.50 – 1.36)	0.453
Model 1	1.11 (0.69 – 1.78)	0.666	1.66 (1.06 – 2.60)	0.025	0.77 (0.46 – 1.28)	0.311
Model 2	1.00 (0.62 – 1.63)	0.987	1.65 (1.06 – 2.57)	0.028	0.92 (0.55 – 1.54)	0.739
Model 3	0.79 (0.47 – 1.33)	0.380	1.47 (0.92 – 2.35)	0.105	0.94 (0.55 – 1.61)	0.816
Model 4	0.79 (0.47 – 1.34)	0.383	1.48 (0.93 – 2.37)	0.099	0.93 (0.54 – 1.59)	0.792
**Nonatherosclerotic CVD mortality**						
Unadjusted	0.67 (0.37 – 1.21)	0.187	0.99 (0.59 – 1.65)	0.957	1.07 (0.65 – 1.75)	0.787

Model 1	0.55 (0.30 – 0.99)	0.048	0.83 (0.50 – 1.38)	0.473	1.04 (0.63 – 1.72)	0.884
Model 2	0.55 (0.30 – 1.02)	0.058	0.80 (0.48 – 1.34)	0.393	1.05 (0.63 – 1.74)	0.865
Model 3	0.50 (0.26 – 0.98)	0.042	0.74 (0.42 – 1.30)	0.298	1.08 (0.63 – 1.83)	0.787
Model 4	0.50 (0.26 – 0.98)	0.042	0.74 (0.42 – 1.30)	0.299	1.08 (0.63 – 1.83)	0.786

	**HR (95% CI)**	***P* value**	**HR (95% CI)**	***P* value**	**HR (95% CI)**	***P* value**
**All-cause mortality**						
Unadjusted	1.51 (1.19 – 1.91)	0.001	1.32 (1.04 – 1.69)	0.023	1.09 (0.85 – 1.39)	0.509
Model 1	1.29 (1.02 – 1.65)	0.037	1.22 (0.96 – 1.56)	0.111	0.96 (0.75 – 1.22)	0.735
Model 2	1.26 (0.98 – 1.60)	0.067	1.20 (0.94 – 1.54)	0.141	1.03 (0.80 – 1.32)	0.825
Model 3	1.10 (0.85 – 1.43)	0.463	1.11 (0.85 – 1.44)	0.439	1.01 (0.78 – 1.32)	0.915
Model 4	1.11 (0.85 – 1.43)	0.450	1.12 (0.86 – 1.45)	0.409	1.01 (0.78 – 1.31)	0.925

Note: We indicated the third quartile (Q3) as the reference group (n = 382).

Abbreviations: CI, confidence interval; CVD, cardiovascular disease; HDL-C, high-density lipoprotein cholesterol ratio; HR, hazard ratio; Q1 to Q4, lowest to highest quartile; SD, standard deviation; SHR, subdistribution hazard ratio.

Model 1: Adjusted for age and sex.

Model 2: Adjusted for model 1 plus diabetes, prior CVD events, body mass index, and systolic blood pressure.

Model 3: Adjusted for model 2 plus hemoglobin, serum albumin, hypersensitive C-reactive protein, and estimated glomerular filtration rate.

Model 4: Adjusted for model 3 plus statin use.
